# Microdialysis of Voriconazole and its *N*-Oxide Metabolite: Amalgamating Knowledge of Distribution and Metabolism Processes in Humans

**DOI:** 10.1007/s11095-022-03407-7

**Published:** 2022-10-21

**Authors:** Josefine Schulz, Robin Michelet, Markus Zeitlinger, Gerd Mikus, Charlotte Kloft

**Affiliations:** 1 Department of Clinical Pharmacy & Biochemistry, Institute of Pharmacy, Freie Universitaet Berlin, Kelchstraße 31, 12169 Berlin, Germany; 2 Department of Clinical Pharmacology, Medical University of Vienna, Waehringer Guertel 18-20, 1090 Vienna, Austria; 3 Department of Clinical Pharmacology and Pharmacoepidemiology, University Hospital Heidelberg, Im Neuenheimer Feld 410, 69120 Heidelberg, Germany

**Keywords:** drug metabolism, microdialysis, pharmacogenetics, pharmacokinetics, voriconazole

## Abstract

**Purpose:**

Voriconazole is an essential antifungal drug whose complex pharmacokinetics with high interindividual variability impedes effective and safe therapy. By application of the minimally-invasive sampling technique microdialysis, interstitial space fluid (ISF) concentrations of VRC and its potentially toxic *N*-oxide metabolite (NO) were assessed to evaluate target-site exposure for further elucidating VRC pharmacokinetics.

**Methods:**

Plasma and ISF samples of a clinical trial with an approved VRC dosing regimen were analyzed for VRC and NO concentrations. Concentration-time profiles, exposure assessed as area-under-the-curve (AUC) and metabolic ratios of four healthy adults in plasma and ISF were evaluated regarding the impact of multiple dosing and CYP2C19 genotype.

**Results:**

VRC and NO revealed distribution into ISF with AUC values being ≤2.82- and 17.7-fold lower compared to plasma, respectively. Intraindividual variability of metabolic ratios was largest after the first VRC dose administration while interindividual variability increased with multiple dosing. The CYP2C19 genotype influenced interindividual differences with a maximum 6- and 24-fold larger AUC_NO_/AUC_VRC_ ratio between the intermediate and rapid metabolizer in plasma and ISF, respectively. VRC metabolism was saturated/auto-inhibited indicated by substantially decreasing metabolic concentration ratios with increasing VRC concentrations and after multiple dosing.

**Conclusion:**

The feasibility of the simultaneous microdialysis of VRC and NO *in vivo* was demonstrated and provided new quantitative insights by leveraging distribution and metabolism processes of VRC in humans. The exploratory analysis suggested substantial dissimilarities of VRC and NO pharmacokinetics in plasma and ISF. Ultimately, a thorough understanding of target-site pharmacokinetics might contribute to the optimization of personalized VRC dosing regimens.

**Supplementary Information:**

The online version contains supplementary material available at 10.1007/s11095-022-03407-7.

## Introduction

The increasing global prevalence of invasive fungal infections is a rising, but underappreciated threat to the public health. Populations at risk of severe fungal infections include patients undergoing allogeneic stem-cell transplantation, and patients with immunosuppressive therapy [1–4]. As this susceptible population is expanding and the prescribing of antifungal agents increasing, resistance to antimycotics poses a growing challenge [3, 5–7]. Yet, the development and market authorization of new antimycotics is lagging behind this epidemiological threat [8]. Consequently, sustaining the effectiveness of frequently used antimycotics is more relevant than ever [3]. Voriconazole (VRC), a second-generation, broad-spectrum triazole antifungal agent, is regularly applied in adults and children as first-line therapy of aspergillosis, candidemia in non-neutropenic patients, as well as for prophylaxis in immunocompromised patients [9–12]. Therapy with VRC in adults is initiated by two intravenous (i.v.) loading doses of 6 mg/kg body weight on day one and continued with two maintenance doses of 4 mg/kg i.v.. As bioavailability is supposed to be high, a switch from i.v. to 200 mg p.o. flat dosing is assumed to be feasible [9]. Although VRC has been on the European and US American drug market for approximately 20 years, knowledge gaps regarding its pharmacokinetic (PK) properties remain [13]. As a result, in clinical practice large intra- and interindividual variabilities are observed, which often lead to adverse drug reactions or therapy failures [14–20]. The main source of variability is assumed to derive from the extensive and complex metabolism of VRC involving the cytochrome P450 (CYP) isoenzymes 2C19, 2C9 and 3A4. The formation of the major circulating metabolite, voriconazole *N*-oxide (NO), is assumed to be substantially dependent on the polymorphic CYP2C19 [13, 21–24]. Although no antifungal activity was demonstrated for NO, it received some attention as potential inducer of adverse reactions, such as photosensitivity and photocarcinogenicity, linked to VRC treatment [25–28].

The target site for anti-infective drugs, also for VRC, is the interstitial space fluids (ISF) as pathogens commonly reside in extravascular spaces [29, 30]. Nevertheless, most PK analyses focus on total plasma concentrations and base dosing recommendations during Therapeutic Drug Monitoring (TDM) on them [31, 32]. However, a complete equilibration between plasma and ISF cannot be assumed without proof [33, 34] and led to recommendations by regulatory agencies to assess target-site concentrations in non-homogenate tissue [35, 36]. In this context, the application of the minimally-invasive sampling technique microdialysis represents an excellent method to sample the protein-unbound fraction over time in ISF. Therefore, the microdialysis catheter is inserted into the interstitial space and continuously perfused with the so-called perfusate, usually a physiological-like solution (e.g. Ringer’s solution), at a flow rate of 1-2 μL/min [37–40]. Consequently, drug molecules, that are present in ISF, can diffuse across the selectively-permeable membrane at the tip of the catheter according to the concentration gradient and are collected in the microdialysate. Due to the continued flow of perfusate, an equilibrium is never reached and thus an assessment of a relative recovery needed. For this purpose, retrodialysis is commonly applied [41, 42]. During retrodialysis, a high concentration of drug is added to the perfusate (so-called retroperfusate) resulting in a delivery of drug molecules into ISF and a reduced concentration of the drug in microdialysate (so-called retrodialysate). Assuming that the fraction of delivery is identical to the fraction recovered, ISF concentrations can be determined [41, 43, 44].

Subsequently to the successful feasibility investigations of the simultaneous microdialysis of VRC and NO *in vitro* [45], the clinical feasibility and applicability of this technique was addressed in this study. Aiming at leveraging at the same time knowledge about distribution and metabolism processes of VRC in humans an exploratory PK analysis was performed, investigating differences in plasma and ISF of four healthy adults with different CYP2C19 genotype-predicted phenotypes after single and multiple VRC dosing, administering an approved dosing regimen.

## Material and Methods

### Drugs and Materials

VRC was administered as VFEND^®^ (Pfizer Inc., Vienna, Austria) in the clinical trial. Analytical reference standards of VRC and NO for bioanalysis were purchased from Toronto Research Chemicals (Toronto, Canada). CMA 60 microdialysis catheters (molar mass cut-off 20 kDa, membrane length 30 mm, M Dialysis AB, Sweden) were used, and perfused with Ringer’s solution (RS) (Ringer Lösung ÖAB, “Mayrhofer“, Mayrhofer Pharmazeutika, Austria). CMA 107 *in vivo* pumps (M Dialysis AB, Sweden) ensured a constant flow of perfusate.

### Clinical Trial Design

From 2009 to 2013, a clinical microdialysis study (ClinicalTrials.gov identifier NCT01539330; EudraCT No. 2008-008524-32) was conducted at the Medical University Vienna. The trial was approved by the Ethics Committee of the university, the competent federal authority (BASG) and performed according to the Declaration of Helsinki as amended in Seoul 2008 [46], the ICH guideline for good clinical practice as well as local regulatory requirements at the Department of Clinical Pharmacology, Medical University Vienna, Austria. Healthy male adults were enrolled in the prospective, open-labeled and uncontrolled trial.

### Study Schedule

All volunteers received twice daily an approved VRC standard dosing regimen of 6 mg/kg body weight i.v. as a 2 h infusion on day one, 4 mg/kg i.v. as a 1.3 h infusion on day two and 200 mg tablets on day three and four [9]. Microdialysis catheters were inserted into ISF of the abdominal subcutaneous adipose tissue for a total of four days and microdialysate as well as plasma samples were collected according to a prespecified schedule. After the first, fifth and seventh dose, intensive sampling was performed by taking 16 plasma samples (0, 0.5, 1, 1.5, 1.75, 2, 2.25, 2.5, 3.25, 4.5, 6, 8, 9, 10, 11, 12 h) as well as collecting microdialysate samples in 16 intervals (0-0.5, 0.5-1, 1-1.5, 1.5-2, 2-2.5, 2.5-3, 3-3.5, 3.5-4, 4-5, 5-6, 6-7, 7-8, 8-9, 9-10, 10-11, 11-12 h) after VRC administration. Sparse sampling was performed after the second, third and fourth VRC dose administration taking plasma samples at the end of the infusion (2 h on day 1, 1.3 h on day 2 and 3) and after 12 h as well as collecting microdialysis samples in 0.33 h intervals until the end of the infusion. After the sixth dose (tablet) on day 3, only after 12 h a plasma sample was taken [38].

To individually calibrate microdialysis catheters, retrodialysis was performed twice, subsequently to the third and last (seventh) dosing interval. Therefore, catheters were perfused with RS containing VRC at a concentration of 200 μg/mL and two retrodialysate samples were collected for 15 min after a 10 min equilibration phase. Perfusate and retrodialysate samples were evaluated individually for every volunteer: relative delivery (rD) was assessed by relating the concentration in retrodialysate (C_Retrodialysate_) to the concentration in retroperfusate (C_Retroperfusate_) as presented in Eq.﻿ 1 and assumed equal to RR. Thus, based on rD determinations, concentrations of VRC and NO in ISF (C_ISF_) were determined from microdialysate concentrations (C_μD_) as described in Eq. 2.

1 $$rD,\%=100\%-\left(\frac{C_{Retrodialysate}}{C_{Retroperfusate}}\right)\cdot 100\%$$

2 $${C}_{ISF}=\frac{C_{\mu D}}{rD,\%}\cdot 100\%$$

### Study Population

Plasma and microdialysate samples from four healthy individuals were available and analyzed to simultaneously quantify the concentrations of VRC and NO. The healthy volunteers were male, between 22 and 28 years old and had a body mass index of 20.5 to 23.4 kg/m^2^. Despite their similar demographic characteristics, they all presented a different CYP2C19 genotype-predicted phenotype. One individual was classified as a rapid metabolizer (RM, CYP2C19 *1/*17), one a normal metabolizer (NM, CYP2C19 *1/*1) and two as intermediate metabolizers (IM, CYP2C19 *1/*2 and CYP2C19 *2/*17) according to the Clinical Pharmacogenetics Implementation Consortium (CPIC^®^, [47]). However, for better differentiation, the CYP2C19 *2/*17 genotype will be referred to as a rapid/poor metabolizer (RM/PM).

### Bioanalysis

Concentrations of VRC and NO in plasma and microdialysate were determined using a previously validated LC-MS/MS assay [48]. An Agilent 1290 Infinity II LC system was used with an InfinityLab Poroshell 120 Phenyl Hexyl column (RP, 2.1 × 100 mm, 2.7 μm, Agilent Technologies, Waldbronn, Germany) for chromatography. Methanol and water (both with 0.1% [V/V] formic acid) were combined in a gradient method at a flow rate of 0.350 mL/min and ensured chromatographic separation. Detection was accomplished by an Agilent triple quadrupole MS/MS system (G6495A) with an electrospray ionization source operated in positive ionization mode. For quantification the following transitions were monitored: *m/z 350 → 281* for VRC, *m/z 366 → 224* for NO and *m/z 285 → 193* for diazepam (internal standard). Using only 20 μL and 5 μL of sample volume, the calibration range for VRC and NO was 0.005-5 μg/mL in plasma and 0.004-4 μg/mL for microdialysate, respectively. All analytical runs met the requirements of the EMA guideline on bioanalytical method validation [49] with accuracy being within 100 ± 15% (±20% at the lower limit of quantification) of the nominal concentration and precision ≤15% coefficient of variation (≤20% at the lower limit of quantification).

### Pharmacokinetic Data Analysis for Clinical Study Samples in Plasma and Interstitial Space Fluids

The obtained clinical data of VRC and NO from plasma and microdialysate were subjected to an exploratory PK analysis. Therefore, the four individuals were assessed individually considering their CYP2C19 genotype-predicted phenotype and changes in PK were analyzed with regard to the number of VRC dose administrations. In particular, PK after the first VRC dose administration (first dosing interval on day one, i.e. considered as single dosing) which reflected an i.v. administration, after the fifth dose (fifth dosing interval on day three, i.e. multiple dosing) which reflected the first p.o. dosing and after the seventh and last dose of the clinical trial (seventh dosing interval on day four, i.e. multiple dosing), was evaluated. Further, differences in PK between plasma and ISF were explored: VRC and NO concentration-time profiles were investigated in plasma and ISF. While concentrations of VRC and NO in plasma samples reflected *total* plasma concentrations, concentrations determined in microdialysate samples were transformed to ISF concentrations by application of retrodialysis data, reflecting unbound target-site concentrations. NO concentrations were calculated using VRC retrodialysis as a surrogate based on *in vitro* feasibility investigations [45]. Lastly, mid-time points of the microdialysis sampling intervals were considered for all evaluations regarding time.

As a PK target in clinical practice, the minimum concentration (C_min_) of VRC in *plasma* is typically considered with target concentrations of 1-2 μg/mL (2.86 – 5.73 μmol/L) but not to exceed limits of 4.5-6 μg/mL (12.9 – 17.2 μmol/L) [32]. Consequently, also the attainment of this C_min_ target was explored for each individual. To assess the exposure over time, the area under the concentration-time curve (AUC) was determined for VRC and NO in plasma and ISF after the first, fifth and seventh VRC dose. The AUC was determined by the linear-up/log-down trapezoidal method from time point 0 to the time point of the last positive, i.e. quantified, concentration using the “pkr” package in R and R Studio^®^ [50]. The extent of tissue fluid exposure was furthermore assessed by the ratio of AUC values between ISF and plasma for VRC and NO, respectively.

#### Metabolic Ratios

For the assessment of the observed differences between the four individuals as well as to investigate the extent of metabolism, ratios of the NO and VRC concentrations were evaluated. For this purpose, metabolic ratios of NO to VRC molar concentrations were calculated for every individual plasma and ISF sample and investigated for changes across time and VRC concentration. Additionally, the metabolic ratios of the molar AUC of NO to VRC were explored in relation to the CYP2C19 genotype-predicted phenotype, the respective dosing interval as well as for the different matrices.

## Results

The twice daily administered i.v. doses on day one ranged from 390 to 461 mg and on day two from 260 to 307 mg compared to the lower twice daily flat 200 mg p.o. doses on days three and four. From a total of 212 plasma and 284 microdialysate samples quantified, besides the baseline samples that were taken before VRC administration, only the concentration of one microdialysate sample was below the lower limit of quantification for VRC and NO and was thus not considered in the further analysis. Furthermore, three microdialysate samples from the CYP2C19 RM/PM in the fifth dosing interval were not available due to a necessary catheter replacement. However, no plasma sample was missing.

### Plasma Concentrations of Voriconazole and its *N*-Oxide Metabolite

**Fig. 1:**
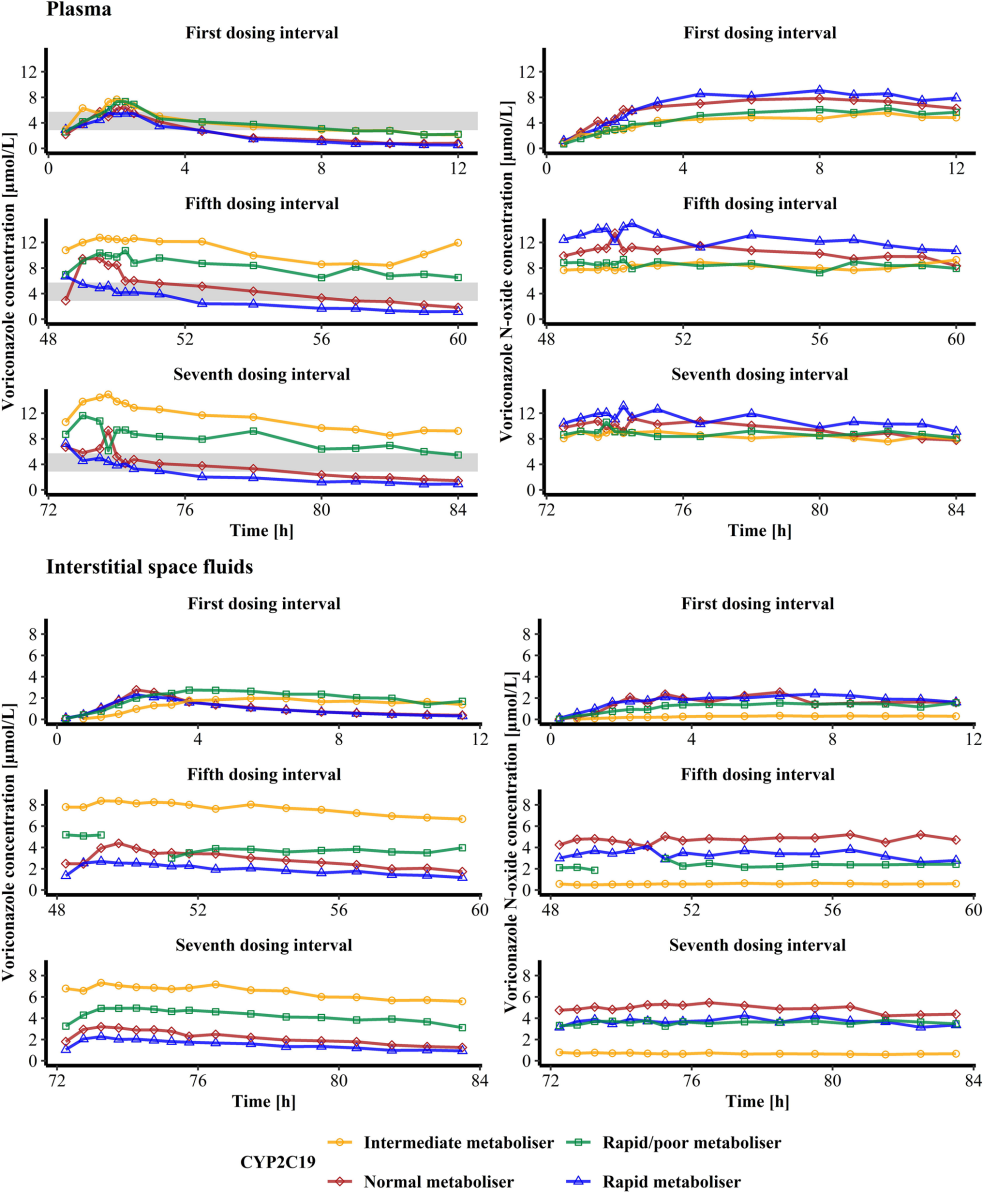
Concentration-time profiles of voriconazole (left) and its *N*-oxide metabolite (right) in plasma (upper panel) and interstitial space fluid (lower panel) in the first, fifth and seventh voriconazole dosing interval of four healthy volunteers with different CYP2C19 genotype-predicted phenotypes. Interstitial space fluid data is represented as mid time point of the sampling interval. The shaded area (upper left) shows the recommended therapeutic target threshold of total voriconazole minimum plasma concentration (2.86 – 5.73 μmol/L) [31, 32].

In total, across all dosing intervals, VRC and NO plasma concentrations ranged from 0.553 to 18.7 μmol/L and from 0.720 to 14.9 μmol/L, respectively. Overall, the CYP2C19 IM showed the highest VRC and lowest NO concentrations, whereas the CYP2C19 RM revealed the lowest VRC and highest NO concentrations, respectively. The interindividual differences between the four individuals substantially increased for VRC plasma concentrations from the first to the last dose, while for NO it was relatively stable (Fig. 1, upper panel).

Maximum plasma concentrations (C_max_, Table S1) of VRC and NO occurred at earlier times (t_max_) after multiple dosing. NO C_max_ after the first VRC dose administration was observed considerably later with a t_max_ of 8 to 10 h, depending on the individual subject.

**Fig. 2:**
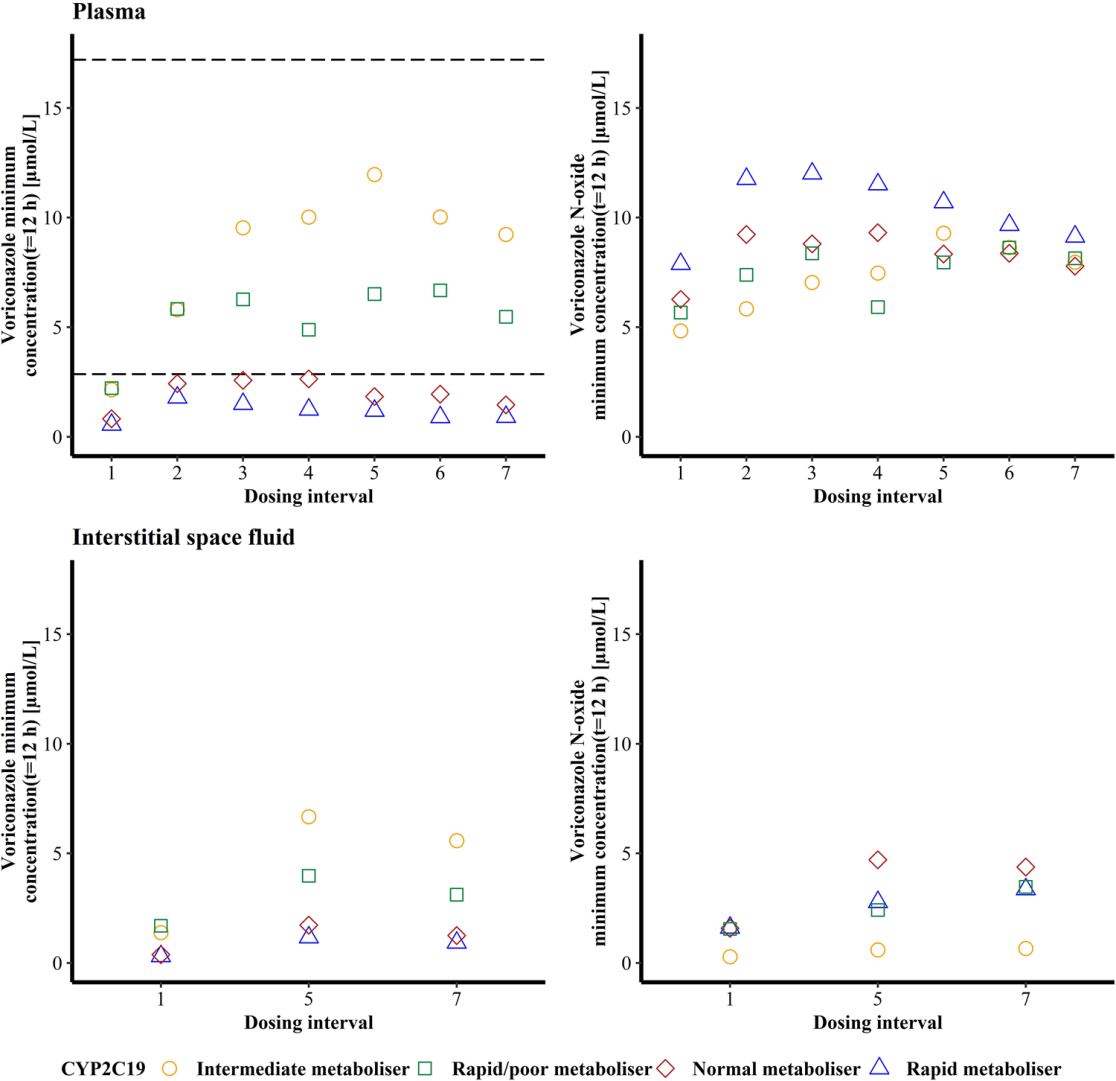
Voriconazole (left) and voriconazole *N*-oxide (right) minimum total plasma (top) and interstitial space fluid (bottom) concentrations determined 12 h after voriconazole dose administration in function of the respective dosing interval (1 to 7) of four healthy volunteers with different CYP2C19 genotype-predicted phenotypes. The dashed lines indicate the recommended therapeutic target range of total voriconazole minimum plasma concentration (2.86 – 17.2 μmol/L) [31, 32].

Regarding the PK target, the lower threshold was not met after the first VRC dose for any of the four volunteers with a maximum C_min_ of 2.22 μmol/L for the RM/PM. After the second VRC dose, the IM and RM/PM exceeded the C_min_ threshold, while the NM and RM did not reach the C_min_ range in any of the dosing intervals. The upper limit of C_min_ of 12.9 – 17.2 μmol/L was never exceeded with a maximum observed C_min_ of 12.0 μmol/L in the CYP2C19 IM after the fifth VRC dose. No PK target range for C_min_ of NO has been defined. In the first dosing interval, C_min_ ranged from 4.83 (IM) to 7.89 μmol/L (RM) and incresead for all individuals until the last i.v. dose before decreasing slightly again until the end of the trial. Overall, a trend was observable, that the interindividual difference between the four individuals of VRC plasma C_min_ increased with multiple dosing, wheareas the interindivudal difference of C_min_ of NO reached the highest level after the second dose before decreasing until the sixth VRC dose administration (Fig. 2, upper panel).

### Distribution into Interstitial Space Fluids

Microdialysate concentrations were transformed to ISF concentrations by taking the mean of both RR determinations of each individual into account, except for the CYP2C19 RM/PM. As the microdialysis catheter of the RM/PM had to be replaced during the fifth dosing interval, RR determinations for the individual catheters were used for ISF concentration determination. Overall RR was high and ranged from 75.2% to 96.8% for the individually performed retrodialysis investigations.

VRC as well as NO showed relevant distribution into ISF. Across all dosing intervals, ISF concentrations ranged from 0.0610 and 10.7 μmol/L for VRC and from 0.0231 to 5.47 μmol/L for NO, respectively. In the first dosing interval the CYP2C19 RM and NM showed very similar concentration-time profiles, reaching C_max_ earlier and revealing a steeper decline in concentrations until the end of the dosing interval than the RM/PM and IM. In the fifth and seventh dosing interval the CYP2C19 IM revealed constantly the highest and the RM the lowest VRC concentrations. For NO the CYP2C19 IM had the lowest concentrations at all times while the RM and NM showed comparable profiles in the first dosing interval before NO concentrations of the NM exceeded those of the RM in the fifth and seventh dosing interval. Similar to observations in plasma, in ISF the interindividual differences of VRC concentrations of the four individuals increased with multiple dosing. However, in ISF this observation was also true for NO (Fig. 1, lower panel).

In ISF, C_max_ (Table S1) of VRC occurred at earlier times after multiple dosing. For NO, t_max_ in ISF was reached in the first dosing interval after 6 – 12 h indicating the time lag for the transformation of VRC. Furthermore, t_max_ of VRC and NO in ISF were similar to t_max_ in plasma, supporting a comparable fast distribution into ISF.

C_min_ of VRC and NO in ISF were available for the first, fifth and seventh dosing interval. Overall, C_min_ of VRC was lower compared to plasma. In the fifth dosing interval, C_min_ of VRC were approximately 2- to 5-fold increased compared to the first dosing interval, before a decrease in the seventh dosing interval occurred (Fig. 2, bottom left panel). For NO, C_min_ in ISF increased 1.6- to 3-fold in the fifth dosing interval compared to the first and continued to rise by 10%-30% in the seventh dosing interval, except for the CYP2C19 NM whose C_min_ decreased by 7% (Fig. 2, bottom right panel).

### Exposure to Voriconazole and its *N*-Oxide Metabolite in Plasma and Interstitial Space Fluids

VRC exposure in plasma, assessed by AUC, was highest for the CYP2C19 IM and lowest for the RM. In the first dosing interval, the interindividual difference was the lowest with AUC ranging from 25.5 to 45.3 μmol·h/L, a 1.8-fold difference between the RM and IM. In the fifth and seventh dosing interval, the interindividual difference increased considerably with AUC of VRC ranging from 31.9 to 124 μmol·h/L and 27.2 to 130 μmol·h/L, respectively, a 3.9- and 4.8-fold lower VRC exposure for the IM compared to the RM. The NO exposure pattern in plasma was vice versa: the highest AUC were determined for the RM and the lowest for the IM. In contrast to VRC, the interindividual difference remained relatively constant across the different dosing intervals. In the first, fifth and seventh dosing interval AUC of NO in plasma ranged from 50.4 to 84.3 μmol·h/L, 96.9 to 146 μmol·h/L and 99.0 to 128 μmol·h/L, respectively. Despite the increase in exposure, the interindividual differences between the four individuals were comparable with a 1.7-, 1.5- and 1.3-fold difference between the RM and IM, respectively.

In ISF, for all individuals AUC of VRC were lower compared to plasma. The AUC of VRC ranged from 11.2 μmol·h/L (RM) to 22.2 μmol·h/L (RM/PM) in the first dosing interval. In the fifth dosing interval the exposure increased (AUC of 21.8 to 86.7 μmol·h/L) and remained constant in the seventh (AUC of 17.0 to 73.1 μmol·h/L). This translated to a 2.0-, 4.0- and 4.3-fold interindividual difference between the four individuals. For AUC of NO in ISF comparable observations were made: in the first dosing interval AUC of NO ranged from 2.84 to 20.7 μmol·h/L representing a 7.3-fold difference between the CYP2C19 RM and IM. In the fifth and seventh dosing interval, it was the IM showing the lowest AUC of NO with 6.61 and 7.62 μmol·h/L, respectively, and the NM revealing the highest AUC of NO with 54.4 and 55.8 μmol·h/L, respectively. This translated to an 8.2- and 7.3-fold higher exposure of NO in the RM in comparison to the IM.

Lastly, the distribution of VRC and NO into ISF was investigated by evaluating the AUC ratios of VRC and NO in ISF to their AUC in plasma. Overall, VRC showed a good distribution to ISF with observed exposure in ISF ranging from 35.5% to 69.8% compared to plasma exposure. Furthermore, multiple dosing led for most individuals to an increase in the ratios. The interindividual differences in tissue penetration were minor (Table I). For NO the distribution into ISF was not as distinct as for VRC. In particular the CYP2C19 IM revealed low ratios and protruded from the other individuals (Table I).

**Table I:** Distribution of voriconazole and voriconazole *N*-oxide into interstitial space fluid (ISF) of four healthy individuals assessed as ratios of area under the concentration time curve (AUC) in ISF to plasma in the three dosing intervals with intensive sampling

CYP2C19 metabolizer	AUC_ISF_/AUC_Plasma_		
	Dosing interval		
	1	5	7
Voriconazole			
Rapid metabolizer	0.440	0.682	0.624
Normal metabolizer	0.425	0.620	0.601
Rapid/poor metabolizer	0.506	0.464	0.524
Intermediate metabolizer	0.355	0.698	0.564
Voriconazole *N*-oxide			
Rapid metabolizer	0.245	0.262	0.329
Normal metabolizer	0.242	0.443	0.494
Rapid/poor metabolizer	0.243	0.270	0.397
Intermediate metabolizer	0.0564	0.0683	0.0769

### Metabolic Ratios

**Fig. 3:**
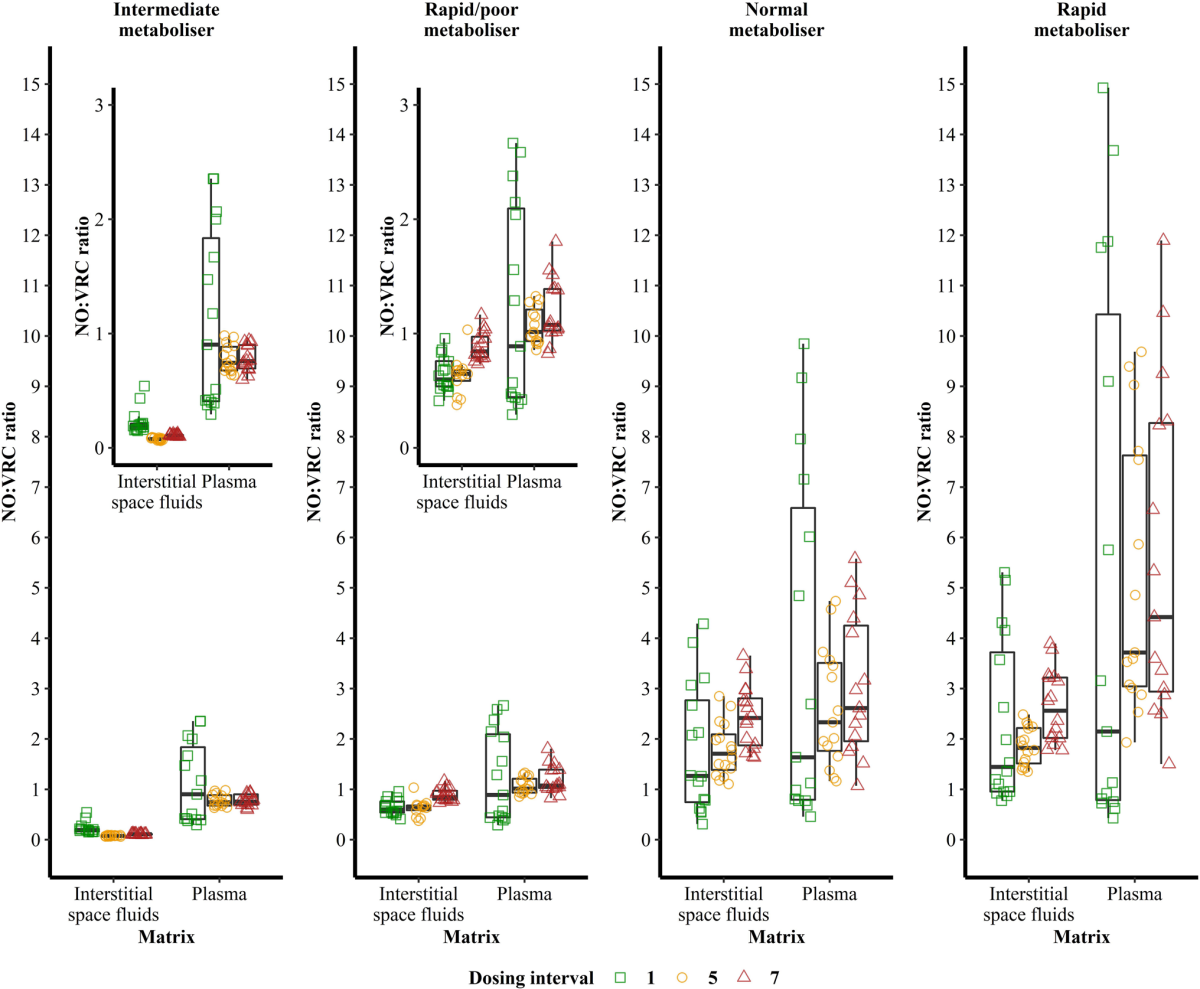
Variability of metabolic ratios of voriconazole *N*-oxide (NO) to voriconazole (VRC) concentrations in interstitial space fluid and plasma in dependence of the CYP2C19 genotype-predicted phenotype of four healthy individuals after the first, fifth and seventh VRC dose administration.

The individual ratios of NO to VRC molar concentrations in plasma showed the largest spread after the first VRC dose administration, decreased for all individuals in the fifth dosing interval, before rising slightly again in the seventh dosing interval. Continued VRC dosing led to an increase in the median metabolic ratios in all CYP2C19 genotypes, except for the IM whose median metabolic ratio was highest after the first VRC dose (Fig. 3). In ISF, a similar pattern was observable: individual metabolic ratios showed the largest variability after the first VRC dose administration, decreased in the fifth dosing interval and rose again after the final VRC p.o. dose administration. The only exception was the CYP2C19 IM who showed a steady decrease in intraindividual variability with continued dosing. The median metabolic ratios were approximately 2-fold lower in ISF compared to plasma for the CYP2C19 RM, NM and RM/PM and > 5-fold decreased for the IM. Multiple dosing resulted in an increase of median metabolic ratios for all individuals in ISF, except for the CYP2C19 IM, who revealed the highest median metabolic ratio in the first dosing interval and the lowest in the fifth (Fig. 3). Overall, the observed difference in metabolic ratios between the four individuals within a matrix was higher in ISF compared to plasma with the median ratios across all dosing intervals being 18- (ISF) and 5-fold (plasma) lower in the CYP2C19 IM compared to the RM, respectively.

During one dosing interval, the individual metabolic ratios of concentrations in plasma and ISF steadily increased, illustrating the time- and concentration-dependent biotransformation of VRC to NO. Furthermore, the CYP2C19 RM showed the steepest increase of metabolic ratios across time (within one dosing interval) and the IM the shallowest. When evaluating the trajectories of metabolic ratio *vs.* time across dosing intervals a flattening in the slope was observed. Overall, this effect was most pronounced for the CYP2C19 RM and NM (Fig. S1).

**Fig. 4:**
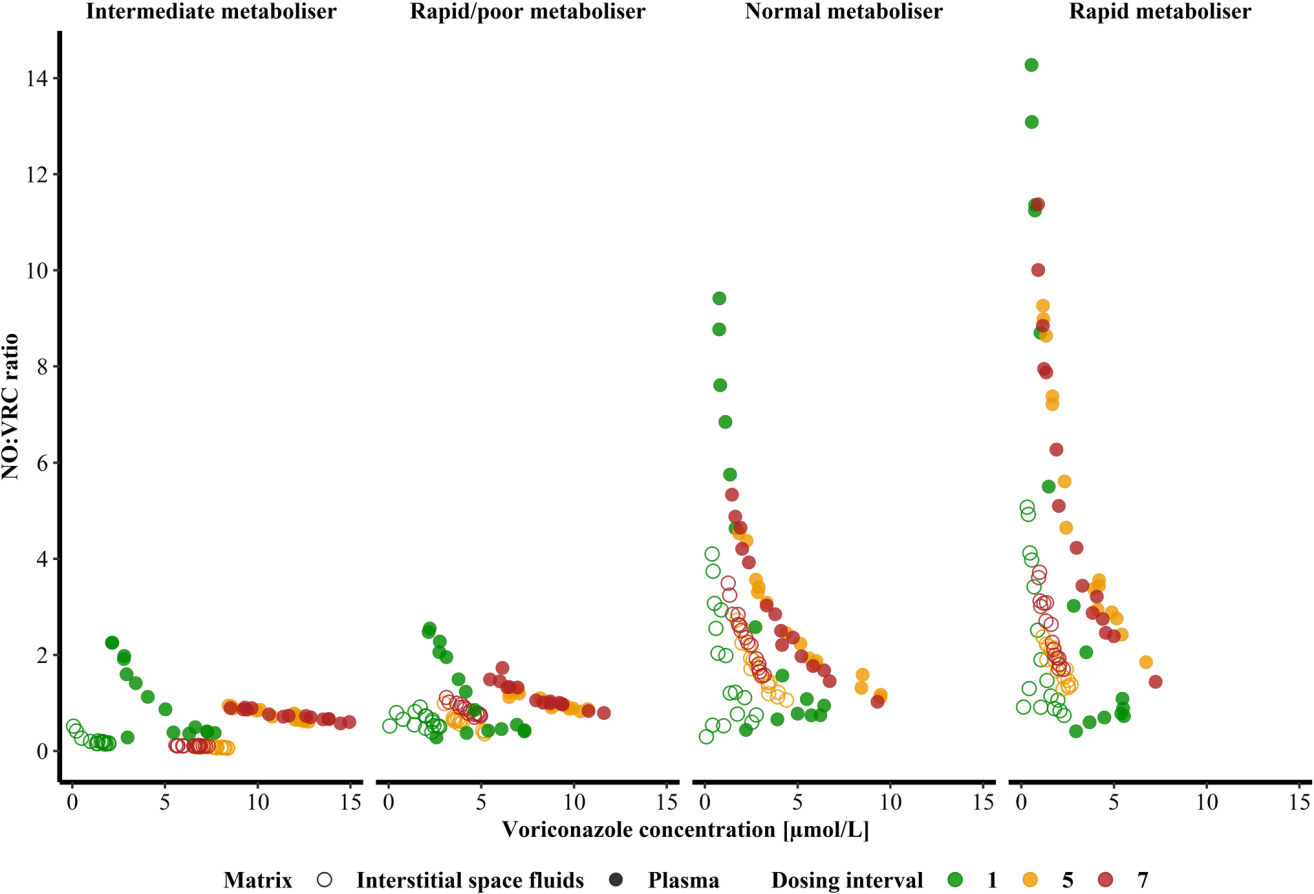
Metabolic ratio of voriconazole *N*-oxide (NO) to voriconazole (VRC) in plasma (filled symbols) and interstitial space fluid (open symbols) in function of the VRC concentration stratified by the CYP2C19 genotype-predicted phenotype of the four healthy volunteers.

The capacity of VRC metabolic transformation to NO was limited, indicated by a decreasing metabolic ratio with increasing VRC concentrations in plasma and ISF within one individual. Independent of the CYP2C19 genotype-predicted phenotype, the decline of the metabolic ratio was non-linear regarding VRC concentration (Fig. 4).

Additionally, the metabolic ratios of the AUC of NO to VRC (AUC_NO_/AUC_VRC_) were assessed in both matrices. In plasma, during the first dosing interval, the interindividual difference between the RM and IM was the smallest with a 3.0-fold range, increased in the fifth dosing interval to a 5.9-fold difference and in the last dosing interval to a 6.2-fold difference in AUC ratios (Table II). In ISF, the differences of AUC ratios between the four individuals were even larger. After the first VRC dose administration, a 10-fold difference between the RM and IM was observed, in the fifth dosing interval it increased to a 23-fold difference and reached a maximum in the seventh and last dosing interval with a 24-fold difference of AUC ratios. In total, AUC ratios in ISF were approximately 2-fold lower than in plasma, except for the IM who revealed a maximum 10-fold difference (Table II).

**Table II:** Ratio of the area under the concentration-time curve (AUC) of voriconazole *N-*oxide (NO) to voriconazole (VRC) in plasma (Left) and interstitial space fluid (Right) of four healthy volunteers with different CYP2C19 genotype-predicted phenotypes in dependence of the respective dosing interval

CYP2C19 metabolizer	AUC_NO_/AUC_VRC_					
	Plasma			Interstitial space fluids		
	Dosing interval			Dosing interval		
	1	5	7	1	5	7
Rapid metabolizer	3.30	4.57	4.71	1.84	1.75	2.49
Normal metabolizer	2.71	2.34	2.78	1.54	1.67	2.28
Rapid/poor metabolizer	1.26	1.04	1.13	0.608	0.604	0.852
Intermediate metabolizer	1.11	0.780	0.764	0.177	0.0763	0.104

## Discussion

In this work, we firstly demonstrated the *in vivo* feasibility and clinical applicability of the simultaneous microdialysis of VRC and its *N*-oxide metabolite in humans. Secondly, the exploratory PK analysis enabled the leveraging of knowledge from target-site distribution and time- and concentration-dependent metabolism processes and highlighted the large intra- and interindividual variability observed in VRC treatment. Thirdly, our results revealed the substantial differences between plasma and ISF PK investigations of VRC and NO. Fourth, the CYP2C19 genotype-predicted phenotype plausibly related to the observed VRC and NO concentrations in plasma and ISF.

Preliminary investigations demonstrated the feasibility of the simultaneous sampling of VRC and NO via microdialysis *in vitro*. Moreover, the analyses showed, that RR of VRC and NO were not significantly different and hence VRC retrodialysis could be applied for the determination of ISF concentrations of both compounds [45]. Therefore, the feasibility and clinical applicability of VRC and NO microdialysis sampling *in vivo* were explored in the current study. Thus, for the first time, the presence of NO in microdialysate samples of a clinical trial were described.

Overall, VRC showed distribution to ISF with observed exposure in ISF ranging from 35.5% to 69.8% compared to plasma exposure between individuals. When the fraction unbound of VRC is considered, which has been reported to amount to 0.50 [51, 52], on average an almost identical exposure of plasma and ISF can be concluded, as only the protein unbound fraction can diffuse unimpededly into tissue extravascular spaces. For NO, the ratios of AUC in ISF and plasma were lower than for VRC, indicating a less distinct distribution of NO to ISF. Potentially also a higher renal excretion of the more hydrophilic metabolite might play a role [53, 54]. This hypothesis is supported by the renal clearances (CL_R_) determined by Geist *et al*. who reported a slightly lower CL_R_ of 1.39 ± 1.04 mL/min for VRC compared to 1.60 ± 1.08 mL/min for NO [15]. Furthermore, NO is more hydrophilic than VRC and carries a charge, characteristics which are typically negatively related to the ability to permeate lipophilic cell membranes. Further reasons for the decreased tissue penetration of NO might be the involvement of efflux transporters or continued (bio)transformation of NO in tissue. Based on the determined AUC the following observations across matrices could be summarized: (i) exposure of VRC and NO was lowest in the first dosing interval, due to accumulation increased in the fifth dosing interval and remained relatively constant in the seventh; (ii) the interindividual difference of AUC of VRC in the four individuals was comparable in plasma and ISF, but was increased in the fifth and seventh dosing interval compared to the first; (iii) the interindividual difference of AUC of NO in the four individuals was relatively constant within one matrix (independent of the dosing interval), but considerably increased in ISF compared to plasma.

Only minor antifungal activity has been described for NO which was described as negligible in the context of VRC treatment [9]. However, due to the large exposure, the *N*-oxide metabolite of VRC might meet current requirements for safety testing according to guidelines of regulatory agencies. This would include toxicity assessments as well as interaction studies for CYP isoenzymes and transporters if the total exposure to the metabolite exceeds certain limits, e.g. *in vitro* CYP interaction studies if the metabolite is more polar than the parent drug and AUC_metabolite_ ≥ AUC_parent_ [55–58]. A comprehensive characterization of NO as a CYP inhibitor has recently been published [24]*.* Additionally, adverse events, such as phototoxicity and photocarcinogenicity observed in patients with long-term VRC treatment, have been related to the metabolite [25–28]. The sensitization of keratinocytes to ultraviolet A (UVA) light by NO and its photoproduct has been shown in *in vitro* experiments [28]. Therefore, distribution of NO into the skin might play an important role, here our results of the distribution of NO into subcutaneous adipose tissue can provide first insights. Currently, only VRC and NO total plasma concentrations have been assessed for their relation to efficacy and/or toxicity that some studies confirmed [59–63] and others did not [27, 62]. In particular, a recent meta-analysis associated the occurrence of neurotoxicity and hepatotoxicity with increased VRC concentrations [63]. Yet, most studies observed high intra- and interindividual variability in VRC concentrations impeding relations to less frequent adverse reactions. In this respect, also the switch in VRC dose administration from a weight-adapted i.v. to a flat p.o. administration is a plausible reason for observed variability. Overall, the assessment to target-site exposure of VRC and NO might lead to a better understanding of its PK relationship to observed pharmacodynamic (PD) effects.

As all four healthy male adult individuals had expectedly very similar demographic characteristics, their CYP2C19 genotype-predicted phenotype was presumably the most influential parameter for the observed interindividual differences [64, 65]. In this context, it was plausible that the RM showed the lowest VRC but highest NO concentrations/exposures while it was reversed for the IM. Interestingly, profiles of the PM/RM were closer to the IM than the RM, suggesting a higher influence of the *2 than the *17 allele on NO formation which justifies the classification of the CPIC^®^ as an intermediate metabolizer [47].

Furthermore, the extent of metabolism was evaluated by the assessment of metabolic ratios. Especially the nonlinearly decreasing metabolic ratios with increasing VRC concentrations were in line with a saturation process of VRC *N*-oxidation or with an onset of inhibition by VRC or a metabolite on its own biotransformation [27, 66, 67]. This could also aid in explaining the observed nonlinear PK of VRC [38, 67]. Additionally, it was shown that the extent of interindividual differences increased when besides plasma VRC concentrations also NO concentrations were considered and that interindividual differences were elevated further when ISF concentrations were examined. In short, VRC and NO plasma concentrations did not represent a reasonable surrogate parameter for target-site pharmacokinetics and disregard important subsequent PK processes that might be essential to assess the appropriateness of VRC dosing regimens. However, when comparing interindividual differences in plasma and ISF, the large method-related variability of microdialysis has to be taken into account. Recent studies thoroughly explored this methodology-related variability and inferred clinical trial recommendations, e.g. determination of individual RR for each patient as well as multiple determinations per patient [68] - settings that were complied with in our study. In a larger and more diverse *patient* population, variability is expected to increase even further, outlining the challenges in safe and effective VRC dosing.

Despite the long storage of the study samples, our investigations yielded very plausible profiles that were comparable to previously reported VRC and NO concentration-time profiles in plasma [15, 17, 66] and ISF [38].

## Limitations

The PK of VRC is highly variable. The presented study with only four healthy individuals each one with a specific CYP2C19 genotype-predicted phenotype must be interpreted as an exploratory approach. Thus, it was not feasible to perform statistical analyses given the number of study participants. A higher variability in PK is expected in patients, e.g. with impaired liver function or infection/inflammation. Both conditions can influence the hepatic metabolism and hence VRC and NO plasma and ISF concentrations. Inflammation is also known to impact the tissue distribution directly, e.g. by increasing the capillary permeation. For confirmation of the generated hypotheses a larger clinical trial with a representative number of individuals and CYP2C19 metabolizer status whose additional medical conditions including concomitant drugs are known is required linking VRC and NO ISF concentrations to efficacy and toxicity.

## Conclusion

The use of microdialysis provides an excellent approach for the determination of unbound drug concentrations in ISF, i.e. the target site of antifungals such as VRC. In our proof-of-concept investigation we demonstrated the feasibility of simultaneous microdialysis of VRC and its *N*-oxide metabolite in humans. Additionally, in an exploratory PK analysis, the clinical applicability of the concomitant observation of VRC and NO in different matrices was shown. The assessment of ISF as well as metabolite concentrations increased the knowledge of the PK of VRC by demonstrating the full extent of interindividual difference. Further research is required to link VRC and NO ISF concentrations to efficacy and toxicity in relation of the CYP2C19 metabolizer status, e.g. by generating a joint drug and metabolite PK model, disentangling the time- and concentration-dependent processes of VRC metabolism as well as VRC and NO target-site distribution, linked to PD parameters. Ultimately, a thorough understanding of the PK of VRC will support the optimization of VRC dosing strategies.

## Supplementary Information

ESM 1 (DOCX 234 kb)
