# Supplementary material for: Microdialysis of Voriconazole and its N-Oxide Metabolite: Amalgamating Knowledge of Distribution and Metabolism Processes in Humans
Source: Pharm Res. 2022 Oct 21;39(12):3279–91. doi: 10.1007/s11095-022-03407-7 (PMC9780129; doi:10.1007/s11095-022-03407-7)
Supplement: Supplementary file 1 — (DOCX 234 kb) [file 11095_2022_3407_MOESM1_ESM.docx]

**Supplement**

**Table S1:** Maximum concentrations of voriconazole and voriconazole N-oxide in plasma and interstitial space fluid of four healthy male adults with different CYP2C19 genotype-predicted phenotypes for the respective dosing interval.

| **CYP2C19 metabolizer** | **Maximum concentration [µmol/L]** | | | | | |
| --- | --- | --- | --- | --- | --- | --- |
|  | **Plasma** | | | **Interstitial space fluids** | | |
|  | **Dosing interval** | | | **Dosing interval** | | |
|  | **1** | **5** | **7** | **1** | **5** | **7** |
| **Voriconazole** |  | | |  |  |  |
| Rapid metabolizer | 5.54 | 6.73 | 7.24 | 2.30 | 2.69 | 2.27 |
| Normal metabolizer | 6.43 | 9.48 | 9.31 | 2.77 | 4.40 | 3.20 |
| Rapid/poor metabolizer | 7.33 | 10.7 | 11.6 | 2.75 | 5.20 | 4.95 |
| Intermediate metabolizer | 7.68 | 12.8 | 14.9 | 1.97 | 8.37 | 7.34 |
| **Voriconazole *N*-oxide** |  | | |  |  |  |
| Rapid metabolizer | 9.09 | 14.9 | 13.1 | 2.37 | 4.12 | 4.24 |
| Normal metabolizer | 7.84 | 13.5 | 11.2 | 2.55 | 5.21 | 5.47 |
| Rapid/poor metabolizer | 6.28 | 9.33 | 10.6 | 1.56 | 2.97 | 3.81 |
| Intermediate metabolizer | 5.54 | 9.29 | 9.19 | 0.338 | 0.651 | 0.791 |


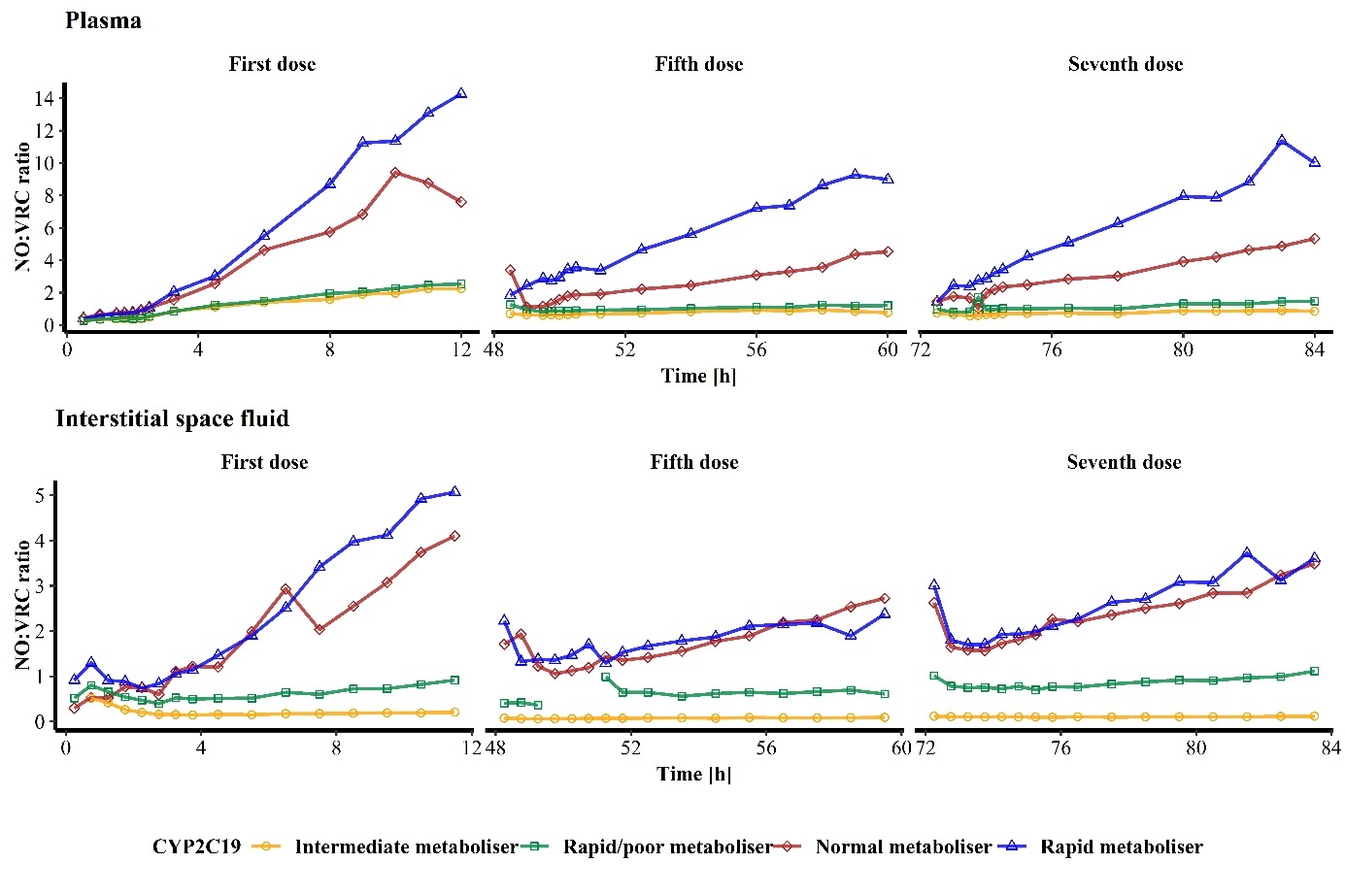


**Figure S1**: Metabolic ratio over time of voriconazole N-oxide (NO) to voriconazole (VRC) concentrations in plasma (upper panel) and interstitial space fluid (lower panel) after the first, fifth and seventh VRC dose administration to four healthy volunteers with different CYP2C19 genotype-predicted phenotypes. Time is presented as hours after the first VRC dose administration.
